# Knowledge-graph-enhanced multi-scale modeling for drug-drug interaction prediction

**DOI:** 10.1016/j.omtn.2026.102855

**Published:** 2026-02-03

**Authors:** Jing Chen, Qiang Deng, Peimeng Zhen, Jialu Hu, Yongtian Wang, Jiajie Peng, Zhuhong You, Xuequn Shang, Xu Zhang, Tao Wang

**Affiliations:** 1 School of Computer Science and Engineering, Xi’an University of Technology, Xi’an 710048, China; 2 School of Computer Science, Northwestern Polytechnical University, Xi’an 710072, China; 3 Key Laboratory of Big Data Storage and Management, Ministry of Industry and Information Technology, Northwestern Polytechnical University, Xi’an 710072, China; 4 Surgery Laboratory, Institute of Medical Sciences, General Hospital of Ningxia Medical University, Yinchuan, Ningxia 750004, China

**Keywords:** MT: Bioinformatics, drug-drug interaction, multi-scale feature fusion, knowledge graph, transformer encoder, deep learning

## Abstract

Drug-drug interaction (DDI) prediction is crucial for understanding combined medication effects and preventing adverse reactions. Traditional machine learning methods rely on handcrafted features and lack generalization, while existing deep learning approaches often fail to capture global and multi-scale drug relationships. To overcome these limitations, we propose ALG-DDI, a multi-scale feature fusion model that integrates three types of drug information: attribute (intrinsic drug structure), local correlations (with proteins and diseases), and global semantic information from the medical knowledge graph PrimeKG. We encode these using attribute masking, the idea of RGCN and GraphSAGE, and ComplEx, respectively. A transformer encoder with attention mechanism then fuses these multi-scale representations. The resulting drug pair vector is fed into a fully connected network for DDI prediction, which we also extend to DDI event prediction. Extensive evaluations on three datasets—including comparative experiments, cross-validation, retrospective analysis, and case studies—demonstrate that ALG-DDI outperforms existing state-of-the-art methods.

## Graphical abstract

**Figure:**
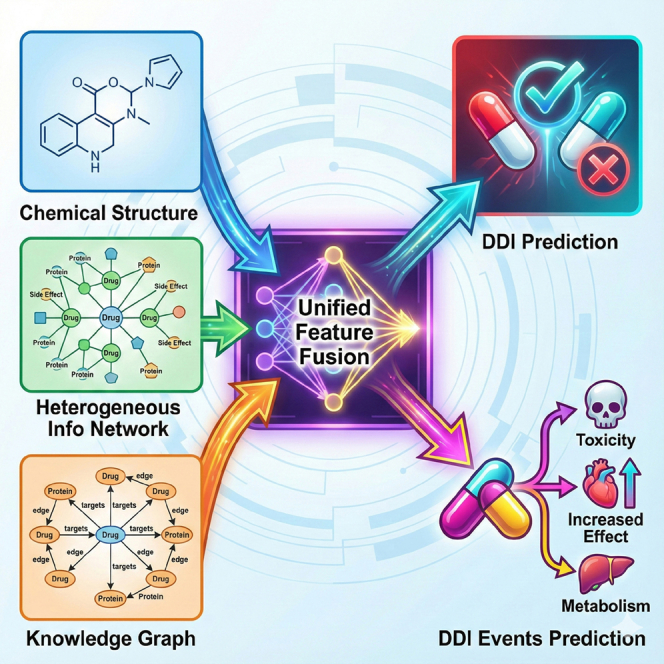

## Teaser

ALG-DDI integrates multi-scale drug features—from structural attributes to global knowledge graph semantics—using a transformer to predict drug-drug interactions. The model outperforms existing methods, offering a generalized, accurate tool for understanding combined medication effects and improving clinical safety.

## Introduction

Drug-drug interactions (DDIs) represent a critical challenge in pharmacotherapy, occurring when multiple medications interact to alter therapeutic effects or induce adverse reactions. ADRs caused by DDIs account for 3%–26% of drug-related hospitalizations,^1^ with recent studies showing 32% of these reactions are preventable.^2^ While traditional experimental methods for DDI detection remain costly and time-consuming, computational methods have emerged as efficient alternatives, evolving through several methodological paradigms.^3^^,^^4^

Early computational approaches to DDI prediction primarily relied on similarity-based methods, building on the fundamental assumption that structurally or functionally similar drugs exhibit analogous interaction patterns. Gottlieb et al. pioneered this approach by integrating multiple similarity measures to generate comprehensive drug features.^5^ Subsequent work by Zhang et al. expanded this paradigm by incorporating 14 distinct similarity measures derived from chemical, biological, phenotypic, and topological data.^6^ More recently, Ryu et al. advanced the field by developing structural similarity profiles processed through deep neural networks,^7^ while Qian et al. demonstrated the effectiveness of combining feature similarity with gradient boosting classifiers.^8^

Parallel developments in matrix factorization techniques have framed DDI prediction as a matrix completion problem. Yu et al. introduced DDINMF, employing semi-non-negative matrix factorization to analyze signed DDI networks.^9^ Zhang et al. further enhanced this approach through manifold regularized matrix factorization, incorporating drug-feature-based regularization.^10^ Zhu et al. devised a dependency network to simulate the relationships between drugs and introduced a probabilistic dependency matrix three-factorization method, known as property-supervised learning model probability dependency matrix three-factorization (PDMTF), for DDI prediction.^11^

The emergence of graph-based methods has represented a significant advancement in DDI prediction, enabling researchers to leverage topological information from various network representations. Initial work by Cami et al. demonstrated the value of network-derived covariates in predictive models.^12^ Subsequent breakthroughs came from Zitnik et al., who formulated DDI prediction as a multi-relational link prediction task using graph convolutional networks.^13^ Recent approaches by Karim et al. and Feng et al. have further refined these techniques through knowledge graph embeddings and specialized graph neural network architectures.^14^^,^^15^

While existing methods have advanced DDI prediction capabilities, they remain fundamentally constrained by their isolated consideration of either local structural features or global network patterns, neglecting the critical interplay between these complementary perspectives. To overcome these limitations, we introduce ALG-DDI, an innovative multi-scale fusion framework that systematically integrates (1) atomic-level molecular attributes extracted from molecular atomic structures, (2) local drug-entity correlations learned from heterogeneous drug association networks, and (3) global semantic patterns derived from biomedical knowledge graphs. The framework’s transformer-based fusion mechanism employs self-attention to dynamically weight and combine these multi-scale representations, enabling comprehensive modeling of drug interactions.

Comprehensive experimental evaluation demonstrates ALG-DDI’s superior predictive performance across multiple benchmark datasets, with rigorous ablation studies quantitatively validating the complementary contributions of each feature scale. Notably, the framework’s successful application to DDI event prediction—a more clinically challenging task—provides compelling evidence of its practical utility in real-world pharmacological scenarios. These results demonstrate the value of multi-scale feature integration for DDI prediction and provide a computational framework that combines molecular-level features with system-level pharmacological information.

## Results

### Comparison of other methods

**Figure 1:**
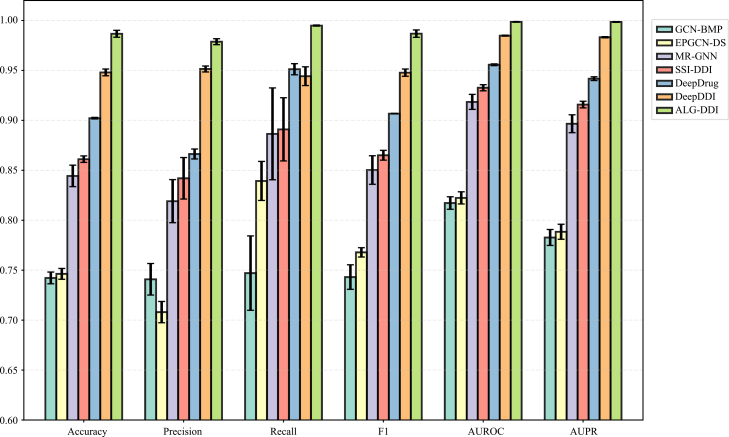
Comparison of DDI prediction performance on DS2 We compared the prediction performance of ALG-DDI with several state-of-the-art methods across six key metrics: accuracy, precision, recall, F1-score, AUROC, and AUPR. Error bars represent the standard deviation of 5-fold cross-validation.

To comprehensively evaluate the performance of ALG-DDI, we compared it with the baseline methods GCN-BMP,^16^ EPGCN-DS,^17^ MR-GNN,^18^ DeepDrug,^19^ SSI-DDI,^20^ DeepDDI,^7^ DDIMDL,^21^ and Lee et al.^22^ To ensure fairness, we uniformly used DS2 as the DDI dataset and reported mean values and standard deviation (sd) based on 5-fold cross-validation as experimental results, which are shown in Figure 1. The specific scores are shown in Table S1. The comparison results indicate that ALG-DDI performs best with outstanding improvements of 3.87%–24.45%, 2.74%–27.07%, 4.37%–24.78%, 3.90%–24.37%, 1.38%–18.14%, 1.53%–21.57%, 0.0107–0.2136, and 0.0123–0.1365 against others in terms of accuracy, precision, recall, F1, ROC-AUC, and PR-AUC, respectively.

We attribute this superior performance to two factors. First, compared to baseline methods that only focus on a single scale, ALG-DDI has a more powerful capability to exploit multi-scale drug information, including the attribute information of drugs themselves, local information from the interactions with key medical entities, and global information from the large-scale knowledge graph. Second, the Transformer encoder based on the self-attention mechanism effectively integrates multi-scale information by assigning dynamic weights, which contributes to its performance.

### Version validation experiment

**Figure 2:**
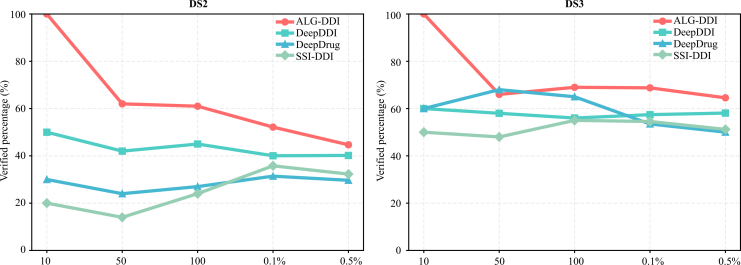
Result of version validation experiment The line chart illustrates the proportion of predicted DDIs from DS1 that were validated in DS2 and DS3.

To further test the efficacy of the model, we conducted a version validation experiment. We trained the model on a lower version of the dataset and made predictions for all other possible DDIs. We selected the top-*N* DDIs based on these scores and verified their existence in higher versions of the dataset. The results are shown in Figure 2. Specifically, we initially compile all possible drug pairs formed by Food Drug Administration (FDA)-approved drugs. The model was trained on DS1 (published in 2014) and used to predict all DDIs absent from DS1 (totaling 7,287,644 pairs). The predictions were then ranked by score, and the top-ranked DDIs were treated as candidates for validation. These candidates were subsequently verified against the newer datasets DS2 (published in 2017) and DS3 (published in 2022) to confirm their occurrence. In this experiment, the top 10, 50, 100, 0.1% (7,291), and 0.5% (36,452) predictions were selected as candidate sets for evaluation. Performance was compared against strong baseline methods, including DeepDDI, DeepDrug, and SSI-DDI.

The results demonstrate that ALG-DDI consistently maintained leading predictive capability. Specifically, all top 10 DDIs predicted by ALG-DDI were subsequently validated in later datasets, whereas the best-performing baseline method validated at most six. Across other proportion thresholds, ALG-DDI achieved the highest validation count in nearly all settings, except for the top 50 predictions on DS3, where DeepDrug validated one more DDI than ALG-DDI. Detailed validation counts are provided in the Table S2.

### Comparison of different fusion strategies

In the feature fusion module of ALG-DDI, we employ the Transformer encoder as a fusion strategy, achieving notable results. To highlight the effectiveness and superiority of the Transformer encoder in our work, we compared it with four common fusion strategies mentioned above. We conducted 5-fold cross-validation experiments on DS1, DS2, and DS3, and the average experimental results are presented in Table 1.

**Table 1:** Performance comparison of four fusion strategies

Dataset	Strategy	Precision	Recall	F1	Accuracy	AUROC	AUPR
DS1	Concat	0.9521	0.9695	0.9606	0.9603	0.9891	0.9852
	Hadamard	0.9034	0.9356	0.9191	0.9177	0.9670	0.9580
	Average	0.9321	0.9651	0.9483	0.9474	0.9827	0.9766
	Transformer	**0.9521**	**0.9857**	**0.9686**	**0.9681**	**0.9899**	**0.9855**
DS2	Concat	0.9736	0.9894	0.9814	0.9813	0.9977	0.9974
	Hadamard	0.9122	0.9289	0.9204	0.9197	0.9721	0.9694
	Average	0.9383	0.9781	0.9578	0.9569	0.9898	0.9879
	Transformer	**0.9788**	**0.9949**	**0.9868**	**0.9867**	**0.9986**	**0.9985**
DS3	Concat	0.9689	0.9828	0.9758	0.9756	0.9975	0.9974
	Hadamard	0.8328	0.8434	0.8380	0.8370	0.9194	0.9166
	Average	0.8956	0.9465	0.9203	0.9181	0.9774	0.9762
	Transformer	**0.9800**	**0.9951**	**0.9875**	**0.9869**	**0.9991**	**0.9990**

This table presents the performance of four fusion strategies across three datasets (DS1, DS2, and DS3) based on six evaluation metrics: precision, recall, F1-score, accuracy, AUROC, and AUPR.

First, we can observe that ALG-DDI demonstrates good robustness, achieving excellent performance on datasets of various sizes. As the size of datasets increases, the results improve even further. Even in DS3, AUROC and AUPR reach the highest values of 0.9991 and 0.9990, respectively. This demonstrates that richer information and more training data enable ALG-DDI to learn more potential patterns, contributing to the improvement of its generalizability.

Additionally, we observed that as the dataset size increases, the advantage of the Transformer encoder becomes more pronounced. The lead in AUROC increases from 0.0008–0.0229 to 0.0016–0.0797. We attribute this to the self-attention mechanism’s ability to allocate weights to different scale features, enabling it to extract more crucial patterns for the task from large-scale data compared to other methods. Surprisingly, we found that the simplest concatenate operation also yields good results. We attribute this to the fact that drug embeddings have already integrated sufficient rich information through the feature extraction module, making straightforward fusion strategies effective as well.

### Model performance on DDI events prediction task

**Figure 3:**
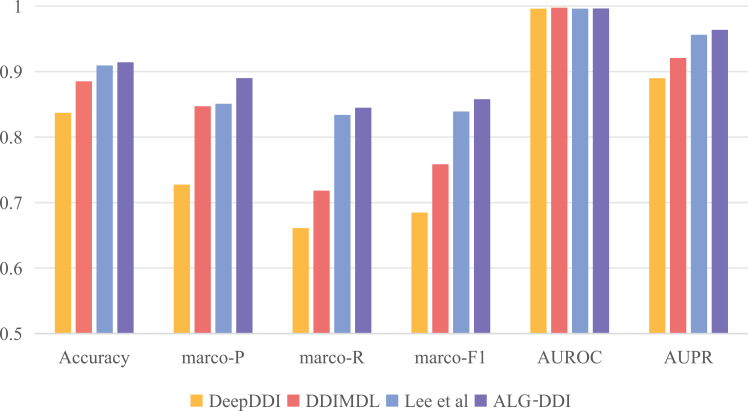
Comparison of DDI events prediction performance on multi-DS We compared the prediction performance of ALG-DDI with several state-of-the-art methods across six key metrics: accuracy, marco-P, marco-R, marco-F1, AUROC, and AUPR.

To comprehensively assess the performance of ALG-DDI, we conducted a series of experiments focused on the DDI event prediction task. In this process, we employed multiple evaluating metrics, including *macro*-*P*, *macro*-*R*, *macro*-*F*1, Accuracy, AUROC, and AUPR. We compared ALG-DDI with other state-of-the-art models, including best-performing baseline DeepDDI in DDI prediction tasks, DDIMDL, and Lee et al., which are specifically designed for DDI event prediction. The average results of 5-fold cross-validation under the same experimental conditions are presented in Figure 3. The detailed scores are shown in Table S3. The confusion matrix, which provides an intuitive visualization of prediction accuracy for each class, is shown in Figure S1.

### Ablation experiments

**Figure 4:**
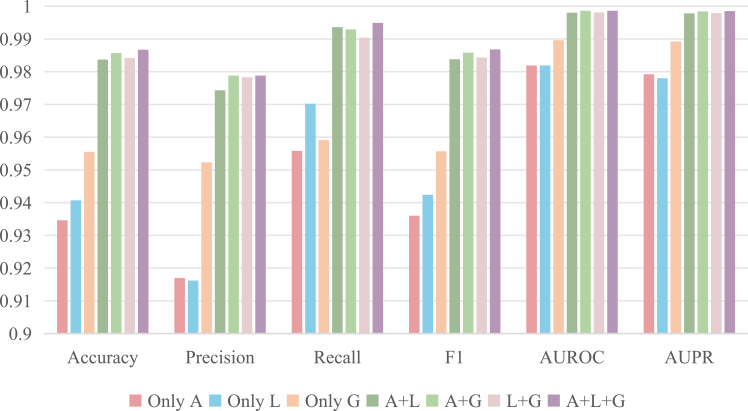
Performance comparison of different feature scale selections on DS2 We compared the performance across six evaluation metrics—accuracy, precision, recall, F1-score, AUROC, and AUPR—under three scenarios: using a single scale, any two scales, and all three scales.

To validate the necessity and contribution degree of information on each scale, we designed six different variants for the ablation study, with three of them using single-scale drug information for experimentation and the other three using information from dual-scale. The results are presented in Figure 4, and the specific scores are shown in Table S4.

First, when we use information from all scales, the model achieves the best performance. Reducing any of the scales results in a performance drop, and employing dual-scale information is more effective than using a single scale alone. These findings strongly demonstrate the necessity and effectiveness of multi-scale feature fusion, indicating that ALG-DDI can indeed enhance the performance through multiple information complementing.

Additionally, we observed that in single-scale experiments, using global information (G) yielded the best results, and in dual-scale experiments, the performance drop was most significant when not using only global information compared to using all scales (A + L + G). This implies that the impact of global information extracted from the knowledge graph is greater compared to other scales. It indicates the correctness of introducing the knowledge graph into the DDI prediction task.

### Case study: Cannabidiol

To validate the excellent performance of ALG-DDI in practical prediction tasks, we conducted a case study using Cannabidiol (CBD). Specifically, we trained the model with the latest DS3 dataset and then scored all unknown drug pairs involving Cannabidiol that were not present in DS3. Higher scores indicate a greater likelihood of a relationship with Cannabidiol. The drugs with the top 15 predicted scores are shown in Table S5, where 14 of them have been further confirmed and included in the DrugBank database, along with detailed DDI descriptions.

To provide a biomedical speculation for the potential interaction between unconfirmed Cannabidiol-Goserelin, we consulted their pathophysiological knowledge. Cannabidiol has anti-tumor effects, which induce cell death pathways, cell growth arrest, and inhibition of tumor angiogenesis, invasion, and metastasis.^23^ Goserelin is a gonadotropin-releasing hormone (GnRH) analogue frequently utilized in the management of hormone-related tumors, including breast cancer and prostate cancer.^24^ Although their mechanisms of action differ, both can act on the relevant symptoms of breast cancer, and in certain situations, they may impact aspects like cancer cell growth, inflammatory responses, or cell apoptosis through different pathways. The relevant study by Alsherbiny et al. also suggests that the cytotoxic mechanisms of CBD are encouraging, providing an impetus for further research on the interaction between CBD and hormonal therapies, including aromatase inhibitors and new-generation drugs such as goserelin.^25^

## Discussion

In this article, we introduced a model named ALG-DDI, which leverages a Transformer encoder to integrate multi-scale drug information for DDI prediction tasks, including attribute information from molecular graphs, local information from heterogeneous graphs, and global information from knowledge graphs. Experimental results have proved that our proposed model is better than state-of-the-art models. In addition, we demonstrated the effectiveness and robustness of our model by conducting experiments across various datasets and employing different fusion strategies. The ablation study also demonstrated the necessity of each scale in feature fusion. In summary, ALG-DDI is an effective model for discovering potential DDIs, which can be used for preliminary screening of potential DDIs before wet laboratory experiments. In the future work, we aim to further optimize the model and bring the idea of multi-scale drug information fusion to the DDI events prediction task, with the expectation of achieving good results.

## Materials and methods

### Datasets

We utilized three key data sources in our study: DrugBank for molecular attributes, PrimeKG for biomedical relationships, and three carefully curated DDI datasets for evaluation.

DrugBank provides comprehensive biochemical and pharmacological information, including drug mechanisms and targets.^26^ We extracted SMILES strings of FDA-approved drugs from the latest DrugBank release for molecular attribute extraction.

PrimeKG is a precision medicine knowledge graph integrating 20 high-quality biomedical resources, containing 129,375 entities and 8,100,498 relationships.^27^ From PrimeKG, we extracted drug-disease, drug-side effect, and drug-protein associations to construct bipartite graphs for local relationship modeling.

To evaluate model performance across different scales, we compiled three DDI datasets.

• DS1: derived from DrugBank 4.0 (2014 release)^26^
• DS2: extracted from DrugBank 5.0 (2018 release), containing ∼6× more interactions than DS1^28^
• DS3: consisting of DDI data from PrimeKG, with remaining PrimeKG data (KGD) reserved for global knowledge extraction

All datasets were rigorously filtered to include only FDA-approved drugs, preventing label leakage between training and knowledge graph sources. Complete dataset statistics are provided in Table 2.

**Table 2:** Summary of dataset details

Dataset	Source	Nodes	Edges
DS1	DrugBank 4.0	1294	80,866
DS2	DrugBank 5.0	1637	392,553
DS3	PrimeKG	2095	1,202,514
KGD	PrimeKG	127,992	5,427,870

### Model architecture

**Figure 5:**
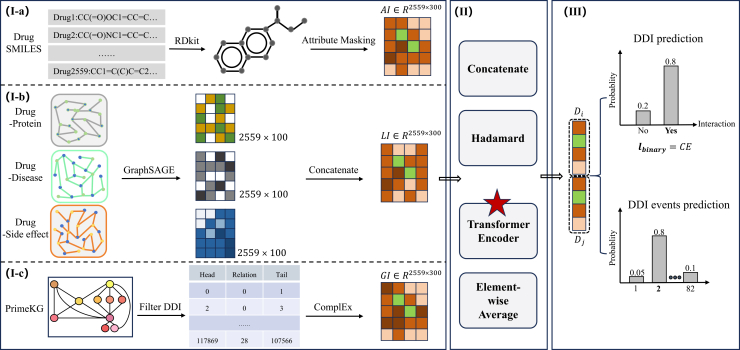
The workflow of ALG-DDI Multi-scale drug feature extraction module (part I): this module extracts drug features at three distinct scales, including (I-a) drug attribute information, (I-b) drug local information, and (I-c) drug global information. Feature fusion module (part II): in this module, a Transformer encoder is employed to integrate features from the three scales to obtain a comprehensive and information-rich final representation. Prediction module (Part III): based on the fused representations of drug pairs, this module predicts both the regulatory relationships and the regulatory relationship categories using a neural network.

Figure 5 illustrates the overall architecture of ALG-DDI, which comprises three main components: (1) multi-scale drug feature extraction, (2) feature fusion, and (3) prediction modules.

1. Multi-scale feature extraction: the framework captures drug representations at three complementary scales:
  • *Atomic-level attributes (AI):* extracted from molecular graphs using attribute masking with graph neural network (GNN) followed by average pooling (Figure 5I-a)
  • *Local interactions (LI):* derived from heterogeneous graphs (drug-side effect, drug-disease, and drug-protein) through relational GraphSAGE (Figure 5I-b)
  • *Global semantics (GI):* obtained from PrimeKG using ComplEx knowledge graph embeddings (Figure 5I-c)
2. Feature fusion: a transformer encoder with self-attention mechanisms dynamically weights and combines the multi-scale representations (AI, LI, GI) to generate comprehensive drug embeddings. This fusion process enables the model to capture cross-scale interactions and learn context-aware representations.
3. Prediction module: for each drug pair, we concatenate their fused representations and process them through a three-layer fully connected neural network to predict interaction probabilities. The network architecture consists of an input layer (concatenated drug features), two hidden layers with ReLU activation, and output layer with sigmoid activation.

The following sections provide detailed descriptions of each component.

### Multi-scale feature extraction

#### Atomic-level attribute representation

For each FDA-approved drug *d*_*i*_∈*D*, we begin by converting its SMILES string to a molecular graph *g*_*i*_ = (*V*,*E*) using RDKit,^29^ where *V* represents atoms (nodes) and *E* represents chemical bonds (edges). We employ an attribute masking approach to learn comprehensive molecular representations through a multi-stage process.^30^

First, we initialize node and edge features based on atomic properties and bond characteristics. The molecular graph then undergoes a feature masking procedure where 15% of node features are randomly replaced with special mask values. These masked features are reconstructed through an iterative graph neural network process. At each layer *k*, node embeddings are updated according to the following equations:

(Equation 1) $$\begin{array}{c} h_{v}^{\left(k\right)}=\text{ReLU}\left(\text{ML}P^{\left(k\right)}\left(\sum_{u\in N\left(v\right)\cup \left\{v\right\}}h_{v}^{k-1}+\sum_{e=\left(v,u\right)}h_{e}^{k-1}\right)\right) \end{array}$$

(Equation 2) $$\begin{array}{c} E_{v}^{\left(k,r\right)}=\sigma \left(W^{k,r}\cdot \text{Concat}\left(E_{v}^{k-1},E_{N\left(v\right)}^{\left(k,r\right)}\right)\right) \end{array}$$

where *N*(*v*) denotes the set of neighboring nodes for atom *v*. To enhance the reconstruction of masked node features, we employ a linear prediction layer that learns to recover the original atomic attributes from the masked representations.^31^^,^^32^^,^^33^

Finally, we aggregate all atom-level representations through average pooling to obtain the graph-level embedding:

(Equation 3) $$\begin{array}{c} h_{G}=\text{MEAN}\left(\left\{h_{v}^{\left(K\right)}|v\in V\right\}\right) \end{array}$$

This pooled representation h_*G*_∈R^*d*^ serves as the atomic-level attribute embedding *a*_*i*_ for drug *d*_*i*_, capturing its fundamental molecular characteristics while maintaining permutation invariance.

#### Local interaction representation

The local interaction patterns between drugs and related biomedical entities provide crucial information for DDI prediction. We model these relationships through a heterogeneous graph framework containing three key association types: drug-side effect, drug-protein, and drug-disease interactions, building upon GraphSAGE and RGCN architectures.^34^^,^^35^

For each relation type *r*∈*R* at layer *k*, our propagation mechanism first aggregates neighborhood information through mean pooling: $E_{N\left(v\right)}^{\left(k,r\right)}=\text{MEAN}\left(\left\{E_{u}^{k-1}|u\in N_{r}\left(v\right)\right\}\right)$, where *N*_*r*_(*v*) denotes the set of neighbors connected to node *v* via relation *r*. The aggregated features are then combined with the node’s current representation and transformed through a relation-specific weight matrix *W*^*k*,*r*^∈R^*d*×2*d*^:

(Equation 4) $$\begin{array}{c} E_{v}^{\left(k,r\right)}=\sigma \left(W^{k,r}\cdot \text{Concat}\left(E_{v}^{k-1},E_{N\left(v\right)}^{\left(k,r\right)}\right)\right) \end{array}$$

with *σ* representing the sigmoid activation function.

The model integrates information across all relation types through summation: $E_{v}^{k}=\sum_{r\in R}E_{v}^{\left(k,r\right)}$. This architecture provides several advantages: relation-specific feature transformation through *W*^*k*,*r*^, gradual expansion of the receptive field with increasing *k*, efficient combination of multiple relation types via summation, and preservation of local topological information through neighborhood aggregation.

The final node embedding $E_{v}^{K}$ at layer *K* captures comprehensive local interaction patterns for drug *d*_*i*_, forming its local representation *l*_*i*_∈R^*d*^. This representation integrates multi-relational information while maintaining the distinct characteristics of each association type through the relation-specific transformations and aggregations.

#### Global semantic representation

The PrimeKG knowledge graph provides essential global semantic information for DDI prediction by capturing high-order relationships between drugs and various biological entities (e.g., pathways, molecular functions, and cellular components). These cross-domain associations help overcome limitations in local feature representations and reveal system-level interaction patterns.

We formalize the knowledge graph as *G*_*KG*_ = {(h,*r*,*t*)|h,*t*∈E,*r*∈R}, where E denotes entities and R represents relationship types. Following recent advances in knowledge graph embedding,^36^ we employ the ComplEx model to learn distributed representations.^37^ ComplEx operates in complex vector space, enabling effective modeling of asymmetric relations through low-rank tensor factorization.

The scoring function evaluates triple plausibility by computing

(Equation 5) $$\begin{array}{c} \phi \left(r,h,t\right)=\text{Re}\left(\sum_{k=1}^{K}w_{rk}e_{hk}\bar{e_{tk}}\right) \end{array}=\left\langle \text{Re}\left(w_{r}\right),\text{Re}\left(e_{h}\right),\text{Re}\left(e_{t}\right)\right\rangle +\left\langle \text{Re}\left(w_{r}\right),\text{Im}\left(e_{h}\right),\text{Im}\left(e_{t}\right)\right\rangle +\left\langle \text{Im}\left(w_{r}\right),\text{Re}\left(e_{h}\right),\text{Im}\left(e_{t}\right)\right\rangle –\left\langle \text{Im}\left(w_{r}\right),\text{Im}\left(e_{h}\right),\text{Re}\left(e_{t}\right)\right\rangle$$

where **w**_*r*_, **e**_h_ and *e*_*t*_∈*C*^*K*^ are complex embeddings, $\bar{e_{t}}$ denotes the complex conjugate, and ⟨·⟩ represents the Hermitian product. The model parameters Θ = {**w**_*r*_,**e**_h_,**e**_*t*_} are learned by minimizing the regularized logistic loss:

(Equation 6) $$\begin{array}{c} L=\sum_{i=1}^{N}\log\left(1+\exp\left(-Y_{h_{i},r_{i},t_{i}}\phi \left(r_{i},h_{i},t_{i}\right)\right)\right)+\lambda |\Theta {|}_{2}^{2} \end{array}$$

with $Y_{h_{i},r_{i},t_{i}}\in \left\{-1,1\right\}$ indicating relation existence and *λ* controlling regularization strength. For each FDA-approved drug *d*_*i*_, its global semantic representation *g*_*i*_∈R^*d*^ corresponds to the real component of the learned entity embedding $\text{Re}\left(e_{d_{i}}\right)$. We compared the performance of ComplEx with several other knowledge graph embedding methods, including TransE_l_1_, TransE_l_2_, DisMult, RotatE, and SimplE. As shown in Table S6, the results demonstrate that ComplEx achieves the best overall performance and shows greater advantages on large-scale datasets.

#### Multi-scale feature fusion

To effectively combine information from different scales, we employ a transformer encoder architecture that dynamically weights and integrates atomic-level (*a*_*i*_), local interaction (*l*_*i*_), and global semantic (*g*_*i*_) features. This approach enables the model to learn cross-scale relationships while preserving the unique contributions of each feature type.

#### Attention-based feature integration

The core of our fusion strategy is multi-head self-attention, which learns contextual relationships between different feature scales. Given the concatenated input *X* = Concat(*a*_*i*_,*l*_*i*_,*g*_*i*_)∈R^3*d*^, we compute

(Equation 7) $${\text{head}}_{i}=\text{Attention}\left(XW_{i}^{Q},XW_{i}^{K},XW_{i}^{V}\right)\;\begin{array}{c} =\text{softmax}\left(\frac{XW_{i}^{Q}{\left(XW_{i}^{K}\right)}^{T}}{\sqrt{d_{k}}}\right)XW_{i}^{V} \end{array}$$

(Equation 8) $$\begin{array}{c} X_{multi}=\text{Concat}\left({\text{head}}_{1},...,{\text{head}}_{m}\right)W^{O} \end{array}$$

where $W_{i}^{Q},W_{i}^{K},W_{i}^{V}\in R^{d\times d_{k}}$ are learnable projection matrices for each attention head, and $W^{O}\in R^{md_{k}\times d}$ combines the heads’ outputs. The attention mechanism automatically learns to emphasize the most informative feature scales for each prediction task.

#### Network architecture components

The transformer encoder employs two key stabilization techniques:

1. Residual connections: we add skip connections that bypass each sub-layer (attention and feedforward),^38^ formulated as

(Equation 9) $$\begin{array}{c} X_{out}=\text{SubLayer}\left(X_{in}\right)+X_{in} \end{array}$$

2. Layer normalization: applied before each sub-layer, it normalizes activations across the feature dimension:

(Equation 10) $$\begin{array}{c} \text{LayerNorm}\left(x\right)=\gamma \frac{x-\mu }{\sqrt{\sigma^{2}+\epsilon }}+\beta \end{array}$$

where *μ* and *σ* are the mean and standard deviation of activations, while *γ* and *β* are learnable parameters,^39^ and *ϵ* is a small constant to prevent division by zero.

#### Alternative fusion strategies

We compare our attention-based fusion against three baseline methods:

(Equation 11) $$\begin{array}{c} X_{\text{concat}}=\text{Concat}\left(a_{i},l_{i},g_{i}\right)\in R^{3d} \end{array}$$

(Equation 12) $$\begin{array}{c} X_{\text{Hadamard}}=a_{i}⊙l_{i}⊙g_{i}\in R^{d} \end{array}$$

(Equation 13) $$\begin{array}{c} X_{\text{average}}=\frac{1}{3}\left(a_{i}+l_{i}+g_{i}\right)\in R^{d} \end{array}$$

All fused representations are projected to dimension *d* through a linear layer for fair comparison. The attention mechanism’s advantage lies in its ability to learn context-dependent weightings of different feature scales, rather than using fixed combination rules.

#### DDI prediction

The prediction module processes learned drug representations to estimate interaction probabilities through a two-stage procedure. For each drug pair (*d*_*i*_,*d*_*j*_), we first concatenate their final representations: *X*_*pair*_ = Concat(*x*_*i*_,*x*_*j*_), where $x_{i},x_{j}\in R^{d}$ are the fused feature vectors for each drug. This combined representation is then processed through a multilayer perceptron (MLP) with sigmoid activation: ${\hat{y}}_{ij}=\sigma \left(\text{MLP}\left(X_{pair}\right)\right)$, producing an interaction probability ${\hat{y}}_{ij}\in \left[0,1\right]$.

The model parameters are optimized using binary cross-entropy loss:

(Equation 14) $$\begin{array}{c} L_{\text{binary}}=-\left[y_{ij}{\log\hat{y}}_{ij}+\left(1-y_{ij}\right)\log\left(1-{\hat{y}}_{ij}\right)\right] \end{array}$$

The model parameter sensitivity analysis and computational cost analysis are presented in Figures S2 and S3, respectively.

### DDI event prediction

We evaluate the model’s generalization capability by extending it to predict specific pharmacological interaction types (e.g., increased metabolism and reduced efficacy). The experimental setup begins with Ryu et al.'s dataset,^7^ from which we derive a DDI event dataset (named Multi-DS) through two preprocessing steps: first retaining only FDA-approved drugs, then removing rare interaction categories with fewer than 10 instances. The resulting Multi-DS contains 82 distinct event types across 172,323 DDIs.

To address the significant class imbalance in Multi-DS, we implement two complementary strategies. First, we employ a phased training approach that combines cross-entropy (CE) and focal loss (FL). During the first half of training (*t*<*T*/2), we use standard CE loss:

(Equation 15) $$\begin{array}{c} L_{\text{CE}}=-\sum_{i=1}^{N}y_{i}\;\log\;{y_{i}}^{\prime } \end{array}$$

which provides stable initial learning, where *N* represents the number of DDI event types. For the second phase (*t* ≥ *T*/2), we switch to FL with *γ* = 2:

(Equation 16) $$\begin{array}{c} L_{\text{FL}}=-\sum_{i=1}^{N}{\left(1-{y_{i}}^{\prime }\right)}^{2}y_{i}\;\log\;{y_{i}}^{\prime } \end{array}$$

to focus learning on harder examples and rare classes.

Second, we employ stratified K-fold cross-validation to maintain proportional class representation across all folds. This approach prevents skewed evaluation metrics that could arise from random splitting, which is particularly important given the long-tailed distribution of interaction types in Multi-DS. The stratification ensures each fold contains representative samples from all 82 event categories, yielding more reliable performance estimates.

### Benchmark

To evaluate the effectiveness of our model, we compared ALG-DDI with eight state-of-the-art methods, including GCN-BMP, EPGCN-DS, MR-GNN, DeepDrug, SSI-DDI, DeepDDI, DDIMDL, and Lee et al. In this work, we employed commonly used classification metrics to evaluate the performance of our framework from various angles, including five key metrics: accuracy, precision, recall, F1, ROC-AUC, and PR-AUC. In addition, we also evaluated the performance of ALG-DDI on the DDI events prediction task, so we introduced metrics suitable for multi-class tasks: macro-P (macro-precision), macro-R (macro-recall), and macro-F1 (macro-precision and recall). Please refer to supplemental information for the introduction of methods and metrics.

## Data and code availability

The DrugBank 4.0 and 5.0 data used in this article are available at DrugBank: https://go.drugbank.com/. The PrimeKG data used can be obtained from Harvard Dataverse: https://doi.org/10.7910/DVN/IXA7BM. The source code of ALG-DDI is available at https://github.com/nwpudengqiang/ALG-DDI.

## Acknowledgments

The authors thank the anonymous reviewers for their valuable suggestions. This work has been supported by Ningxia Central Government-guided Local Science and Technology Development Special Project (2024FRD05103), the 10.13039/501100001809National Natural Science Foundation of China (grant no. 62572391 and 62402382), and Special Talent Introduction Project of Ningxia Autonomous Region Key R&D Programs (2023BSB03053).

## Author contributions

T.W. and X.Z. conceived the experiment(s); J.C, Q.D., and P.Z. conducted the experiment(s); J.H., Y.W., J.P., Z.Y., and X.S. analyzed the results. T.W., J.C, Q.D., and P.Z. wrote and reviewed the manuscript.

## Declaration of interests

No competing interest is declared.

## Declaration of generative AI and AI-assisted technologies in the writing process

During the preparation of this work, the authors used Deepseek in order to assist with language polishing and refinement. After using this tool, the authors reviewed and edited the content as needed and take full responsibility for the content of the published article.

## Supplemental information

Document S1. Figures S1–S3 and Tables S1–S6

Document S2. Article plus supplemental information
