# Supplementary material for: Knowledge-graph-enhanced multi-scale modeling for drug-drug interaction prediction
Source: Mol Ther Nucleic Acids. 2026 Feb 3;37(1):102855. doi: 10.1016/j.omtn.2026.102855 (PMC12926637; doi:10.1016/j.omtn.2026.102855)
Supplement: Document S1. Figures S1–S3 and Tables S1–S6 [file mmc1.pdf]

OMTN, Volume *37*

Supplemental information

Knowledge-graph-enhanced multi-scale modeling for drug-drug interaction prediction

Jing Chen, Qiang Deng, Peimeng Zhen, Jialu Hu, Yongtian Wang, Jiajie Peng, Zhuhong You, Xuequn Shang, Xu Zhang, and Tao Wang

Comparison methods

- **GCN-BMP** utilizes the GCN with Bond-aware Message Propagation to encode molecular graphs. And introduce the self-contained attention mechanism to identify crucial local atoms that align with domain knowledge, providing a level of interpretability.
- **EPGCN-DS** proposed a GCN based framework for type-specific DDI identification from molecular structures. The proposed framework includes an encoder with GCN layers and a decoder that captures complicated interactions while preserves permutation invariant on inputs.
- **MR-GNN** is an end-to-end GNN that employs a multi-resolution-based architecture to extract node features from various neighborhoods of each node. Additionally, it utilizes long short-term memory networks (LSTMs) to summarize local features of each graph and extract interaction features between pairwise graphs.
- **DeepDrug** developed a deep learning framework, which can use graph convolutional networks(GCN) to learn graphical representations of drugs and proteins, such as molecular fingerprints and residual structures, to improve prediction accuracy.
- **SSI-DDI** proposed a deep learning framework, which operates directly on the raw molecular graph representations of drugs for richer feature extraction and decomposes the DDI prediction task between two drugs into the identification of pairwise interactions between the substructures of the respective drugs.
- **DeepDDI** designed a multi-class DDI prediction model utilizing a DNN framework. Structural information of each drug in the input drug pair was employed to generate a feature vector referred to as the structural similarity profile, which was designed to effectively capture a drug's unique structural characteristics.
- **DDIMDL** introduces a multimodal deep learning framework for predicting DDI-associated events. It first constructs deep neural network sub-models based on four types of drug features, and then combines these sub-models to learn cross-modal representations between drug pairs.
- **Lee et al.** utilizes autoencoders and a deep feedforward network, trained on the structural similarity profiles, Gene Ontology term similarity profiles, and target gene similarity profiles of known drug pairs, to predict the pharmacological effects of DDIs.

Evaluation metrics

- **Accuracy** evaluates overall correctness and it can be calculated using the following formula: $\mathrm{Accuracy} = (\mathrm{TP} + \mathrm{TN})/(\mathrm{TP} + \mathrm{FN} + \mathrm{FP} + \mathrm{TN})$ where TP, TN, FP and FN indicate the true positive, true negative, false positive and false negative, respectively.
- **Precision** measures how many of the samples predicted as positive by the model are actually true positive cases, rather than false positives. It can be calculated using the following formula: $\mathrm{Precision} = \mathrm{TP}/(\mathrm{TP} + \mathrm{FP})$.
- Macro-precision is calculated in multi-class classification tasks by computing the precision for each class and then taking the average of these precision values: $\mathrm{macro} - \mathrm{P} = \sum_{i=1}^{n} \mathrm{P}_i$.
- **Recall**, also known as sensitivity, measures how many of the actual positive cases the model correctly identifies. It can be calculated using the following formula: $\mathrm{Recall} = \mathrm{TP}/(\mathrm{TP} + \mathrm{FN})$.
- **Macro-recall** is calculated in multi-class classification tasks by computing the recall for each class and then taking the average of these recall values: $\mathrm{marco} - \mathrm{R} = \sum_{i=1}^{n} \mathrm{R}_i$.
- F1 score is a trade-off between precision and recall. And the harmonic mean of average precision and recall score can be calculated using the following formula: $\mathrm{F1} = (2 \cdot \mathrm{Precision} \cdot \mathrm{Recall})/(\mathrm{Precision} + \mathrm{Recall})$. Similarly, $\mathrm{macro} - \mathrm{F1}$ can be calculated using the following formula: $\mathrm{macro} - \mathrm{F1} = (2 \cdot \mathrm{macro} - \mathrm{P} \cdot \mathrm{macro} - \mathrm{R})/(\mathrm{macro} - \mathrm{P} + \mathrm{macro} - \mathrm{R})$.
- **ROC-AUC** is the abbreviation for "Area under the Receiver Operating Characteristic Curve", which represents the area under the plot of the true positive rate against the false positive rate at various thresholds.
- **PR-AUC** is the abbreviation for "Area under the Precision-Recall Curve", which represents the area under the plot of the precision rate against recall rate at various thresholds.

Supplemental Tables

Table S1 The detailed accuracy, precision, recall, F1-score, AUROC, and AUPR scores on DS2.

Model	Precision	Recall	F1	Accuracy	AUROC	AUPR
GCN-BMP	0.7409	0.7471	0.7431	0.7422	0.8172	0.7828
EPGCN-DS	0.7081	0.8394	0.7679	0.7463	0.8224	0.7885
MR-GNN	0.8191	0.8865	0.8503	0.8444	0.9185	0.8966
SSI-DDI	0.8420	0.8910	0.8651	0.8612	0.9327	0.9158
DeepDrug	0.8664	0.9512	0.9068	0.9022	0.9557	0.9418
DeepDDI	0.9514	0.9442	0.9478	0.9480	0.9848	0.9832
ALG-DDI	**0.9788**	**0.9949**	**0.9868**	**0.9867**	**0.9986**	**0.9985**

Table S2 Specific successful validations in Top n under different versions.

Dataset	Methods	10	50	100	0.1% (7291)	0.5% (36452)
DS2	ALG-DDI	**10**	**31**	**61**	**3803**	**16285**
	DeepDDI	5	21	45	2920	14644
	DeepDrug	3	12	27	2290	10812
	SSI-DDI	2	7	24	2609	11766
DS3	ALG-DDI	**10**	33	**69**	**5014**	**23531**
	DeepDDI	6	29	56	4187	21177
	DeepDrug	6	**34**	65	3900	18238
	SSI-DDI	5	24	55	3976	18665

Table S3 The detailed marco-P, marco-R, marco-F1, accuracy, AUROC, and AUPR scores on Multi-DS.

Model	macro-P	macro-R	Macro-F1	Accuracy	AUROC	AUPR
DeepDDI	0.7275	0.6611	0.6848	0.8371	0.9961	0.8899
DDIMDL	0.8471	0.7182	0.7585	0.8852	**0.9976**	0.9208
Lee et al.	0.8509	0.8339	0.8391	0.9094	0.9961	0.9562
ALG-DDI	**0.8901**	**0.8449**	**0.8579**	**0.9142**	0.9964	**0.9637**

Table S4 The detailed precision, recall, F1-score, accuracy, AUROC, and AUPR scores under different feature scale selections on DS2.

Scale	Precision	Recall	F1	Accuracy	AUROC	AUPR
A	0.9170	0.9558	0.9360	0.9346	0.9819	0.9792
L	0.9162	0.9702	0.9424	0.9407	0.9819	0.9780
G	0.9523	0.9591	0.9557	0.9555	0.9896	0.9892
A+L	0.9743	0.9936	0.9838	0.9837	0.9980	0.9978
A+G	0.9788	0.9929	0.9858	0.9857	0.9986	0.9984
L+G	0.9783	0.9904	0.9843	0.9842	0.9981	0.9979
A+L+G	**0.9788**	**0.9949**	**0.9868**	**0.9867**	**0.9986**	**0.9985**

Table S5 The detailed drugs' information with the top 15 predicted scores.

Rank	Drug	Drugbank ID	DDI Describtion
1	Desmopressin	DB00035	The risk or severity of hypertension can be increased.
2	Cyclosporine	DB00091	The serum concentration of Cyclosporine can be increased.
3	Folic acid	DB00158	Cannabidiol may decrease the excretion rate of Folic acid.
4	Fluvoxamine	DB00176	The risk or severity of adverse effects can be increased.
5	Valsartan	DB00177	The metabolism of Cannabidiol can be decreased.
6	Amphetamine	DB00182	The metabolism of Amphetamine can be decreased.
7	Nicotine	DB00184	The metabolism of Nicotine can be decreased.
8	Cevimeline	DB00185	The metabolism of Cevimeline can be decreased.
9	Lorazepam	DB00186	The risk or severity of adverse effects can be increased.
10	Goserelin	DB00014	N.A.
11	Esmolol	DB00187	The metabolism of Esmolol can be decreased.
12	Bortezomib	DB00188	The risk or severity of serotonin syndrome can be increased.
13	Tramadol	DB00193	The serum concentration of Bortezomib can be increased.
14	Betaxolol	DB00195	The metabolism of Betaxolol can be decreased.
15	Sildenafil	DB00203	The metabolism of Sildenafil can be decreased.

Table S6 The impact of different knowledge graph embedding methods on model performance.

Dataset	KGE model	Precision	Recall	F1	Accuracy	AUROC	AUPR
DS1	TransE_l_1	0.9522	0.9832	0.9674	0.9669	0.9897	0.9857
	TransE_l_2	**0.9528**	0.9849	0.9685	0.9680	0.9902	**0.9863**
	DisMult	0.9499	0.9855	0.9674	0.9667	0.9894	0.9848
	RotatE	0.9518	0.9833	0.9673	0.9667	**0.9901**	0.9861
	SimplE	0.9498	**0.9862**	0.9676	0.9670	0.9896	0.9853
	ComplEx	0.9521	0.9857	**0.9686**	**0.9681**	0.9899	0.9855
DS2	TransE_l_1	0.9742	0.9936	0.9838	0.9836	0.9980	0.9978
	TransE_l_2	0.9748	0.9936	0.9841	0.9839	0.9980	0.9978
	DisMult	0.9769	0.9943	0.9855	0.9854	0.9983	0.9981
	RotatE	0.9756	0.9931	0.9842	0.9841	0.9980	0.9978
	SimplE	0.9768	0.9941	0.9854	0.9852	0.9982	0.9981
	ComplEx	**0.9788**	**0.9949**	**0.9868**	**0.9867**	**0.9986**	**0.9985**
DS3	TransE_l_1	0.9737	0.9898	0.9817	0.9816	0.9986	0.9985
	TransE_l_2	0.9749	0.9902	0.9825	0.9824	0.9987	0.9986
	DisMult	0.9758	0.9901	0.9829	0.9828	0.9987	0.9987
	RotatE	0.9741	0.9911	0.9825	0.9824	0.9987	0.9986
	SimplE	0.9742	0.9906	0.9823	0.9822	0.9987	0.9986
	ComplEx	**0.9800**	**0.9920**	**0.9859**	**0.9859**	**0.9991**	**0.9990**

Supplemental Figures

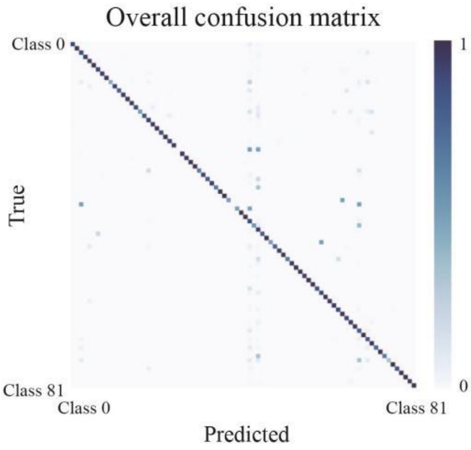

Figure S1 The confusion matrix of DDI events prediction.

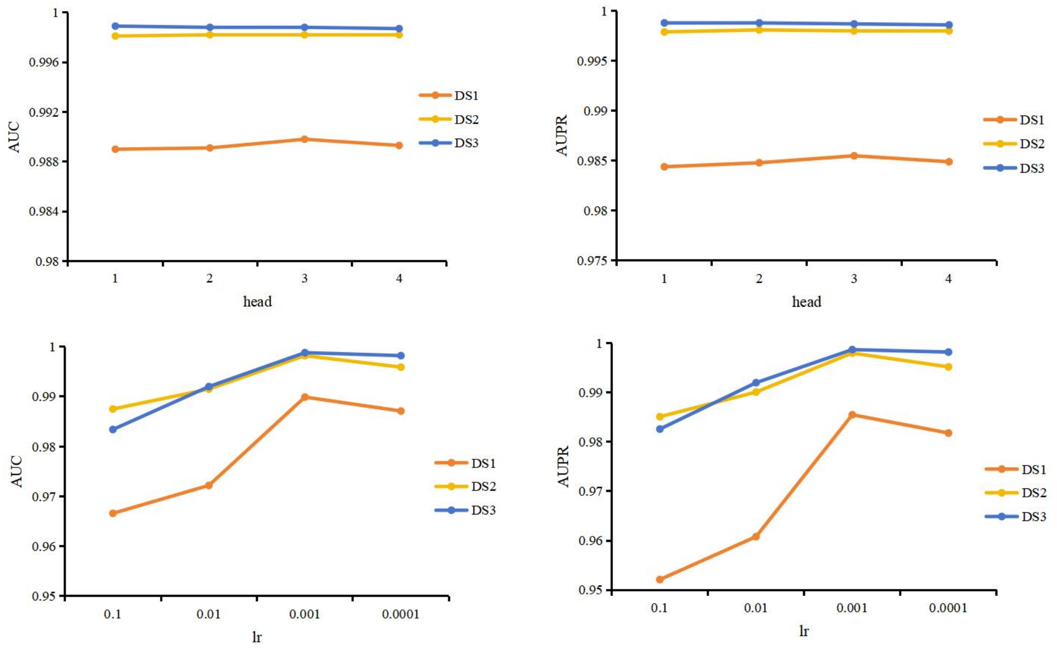

Figure S2 The impact of different attention heads and learning rate on model performance.

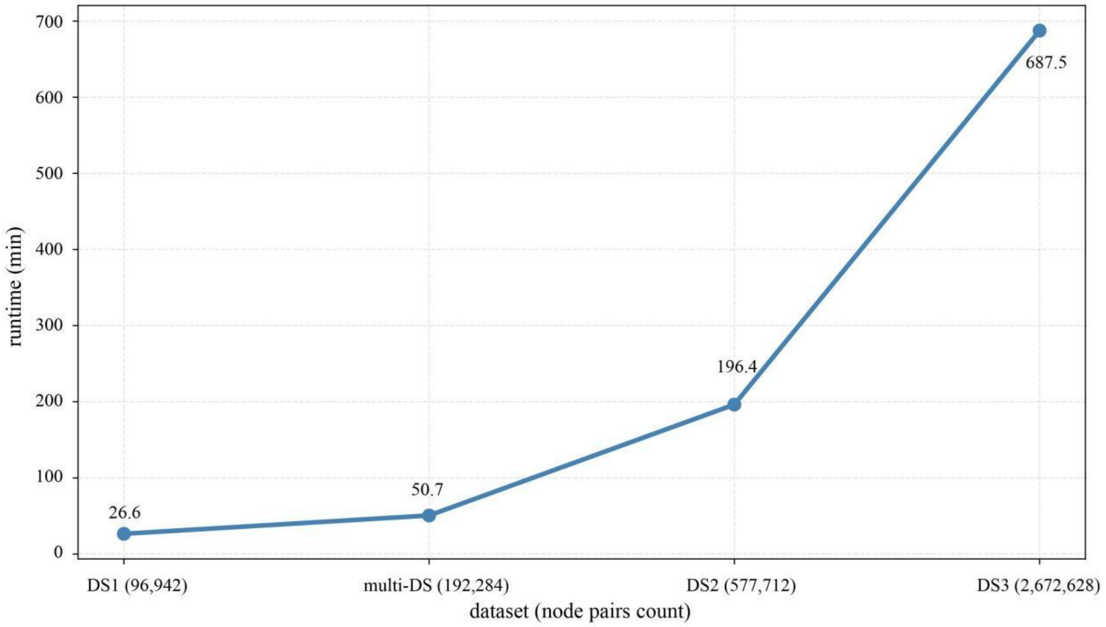

Figure S3 The The time cost of the model on different datasets (data size).
